# Supplementary material for: Molecular Surveillance of Canine Degenerative Myelopathy in Breeding Kennels from Romania
Source: Animals (Basel). 2023 Apr 19;13(8):1403. doi: 10.3390/ani13081403 (PMC10135041; doi:10.3390/ani13081403)
Supplement: Supplementary file 1 [file animals-13-01403-s001.zip › animals-2268773-SI.pdf]

Table S1. Mutant allele frequency obtained in overlapping breeds from the current study and by Zeng et al. [22]

Dog breed	Mutant allele frequency obtained in the current study	Mutant allele frequency obtained by Zeng et al [22]
Wire Fox Terrier	1.0000	0.9400
German Shepherd	0.2000	0.3700
Rottweiler	0.0833	0.0300
Belgian Shepherd	0.0313	0.0600
Czechoslovakian Wolfdog	0.5000	0.3400
Tibetan Mastiff	0.0000	0.0000
Saint-Bernard	0.0000	0.1300
Central Asia Shepherd Dog	0.0000	0.0000
Labrador Retriever	0.0000	0.0700
Italian Cane Corso	0.0000	0.0000
American Staffordshire Terrier	0.0000	0.3300
Beagle	0.0000	0.0600
Bull Terrier	0.0000	0.0000
Staffordshire Bull Terrier	0.0000	0.1100
Flat Coated Retriever	0.0000	0.0500
Dogo Argentino	0.0000	0.0000
Golden Retriever	0.0000	0.0300
Bernese Mountain Dog	0.0000	0.3800
Shar Pei	0.0000	0.0000
American Bulldog	0.0000	0.0500
French Bulldog	0.0000	0.1600
